# Highly Efficient T2 Cobalt Ferrite Nanoparticles Vectorized for Internalization in Cancer Cells

**DOI:** 10.3390/ph14020124

**Published:** 2021-02-05

**Authors:** Eva Mazarío, Magdalena Cañete, Fernando Herranz, Jorge Sánchez-Marcos, Jesús M. de la Fuente, Pilar Herrasti, Nieves Menéndez

**Affiliations:** 1 Departamento de Química Física Aplicada, Facultad de Ciencias, Universidad Autónoma de Madrid, Francisco Tomás y Valiente 7, Cantoblanco, 28049 Madrid, Spain; jorge.sanzhezm@uam.es (J.S.-M.); pilar.herrasti@uam.es (P.H.); nieves.menendez@uam.es (N.M.); 2 Departamento de Biología, Facultad de Ciencias, Universidad Autónoma de Madrid, C/Darwin 2, Cantoblanco, 28049 Madrid, Spain; magdalena.canete@uam.es; 3 Instituto de Química Médica (IQM-CSIC) and CIBER de Enfermedades Respiratorias (CIBERES), Juan de la Cierva 3, 28006 Madrid, Spain; fherranz@iqm.csic.es; 4 Instituto de Nanociencia y Materiales de Aragón, CSIC, Universidad de Zaragoza, C/Pedro Cerbuna 12, 50009 Zaragoza, Spain; jmfuente@unizar.es; 5 Networking Biomedical Research Centre of Bioengineering, Biomaterials and Nanomedicine (CIBER-BBN), 28029 Madrid, Spain

**Keywords:** targeting, contrast agent, cobalt ferrite, nanoparticles, folic acid, internalization

## Abstract

Uniform cobalt ferrite nanoparticles have been synthesized using an electrochemical synthesis method in aqueous media. Their colloidal, magnetic, and relaxometric properties have been analyzed. The novelty of this synthesis relies on the use of iron and cobalt foils as precursors, which assures the reproducibility of the iron and cobalt ratio in the structure. A stable and biocompatible targeting conjugate nanoparticle-folic acid (NP-FA) was developed that was capable of targeting FA receptor positivity in HeLa (human cervical cancer) cancer cells. The biocompatibility of NP-FA was assessed in vitro in HeLa cells using the MTT assay, and morphological analysis of the cytoskeleton was performed. A high level of NP-FA binding to HeLa cells was confirmed through qualitative in vitro targeting studies. A value of 479 Fe+Co mM^−1^s^−1^ of transverse relaxivity (r_2_) was obtained in colloidal suspension. In addition, in vitro analysis in HeLa cells also showed an important effect in negative T2 contrast. Therefore, the results show that NP-FA can be a potential biomaterial for use in bio medical trials, especially as a contrast agent in magnetic resonance imaging (MRI).

## 1. Introduction

Magnetic nanoparticles (NPs) in general have received substantial attention for their theranostic potential activity (ability to combine therapeutic and diagnostic agents within the same device). Some of the most important characteristics in the nanometer regime that can be tunable to optimize the magnetic properties needed for an effective imaging and thermal activation are the saturation of magnetization, coercivity, and magnetocrystalline anisotropy [1]. Among others, ferrite nanoparticles have attracted special attention in the last decades because they are easily synthesizable. There is a great variety of synthesis methods, that are inexpensive and easy to perform, resulting in ferrites with excellent crystallinity and magnetic properties [2,3,4].

The superparamagnetic (SPM) character and large magnetic moments of ferrite magnetic nanoparticles convert them into good candidates to be used as magnetic resonance imaging (MRI) contrast agents, which results from the cooperativity of the individual spins when aligned in the presence of an external magnetic field. SPM nanoparticles perturb the external magnetic field, decreasing the relaxation times (T_1_ or T_2_) by defocusing the magnetization vector (M) ensemble in the precession axis, being T_1_ the relaxation time of M_y_ parallel to the precession axis and T_2_ the relaxation time of the magnetization vector (M_x,y_) in the perpendicular plane to the precession axis. Therefore, these magnetic field perturbations are responsible for the changed relaxivities of the water molecules measured. This produces a darker intensity signal in the case of perpendicular or T_2_ relaxation and a brighter signal for T_1_ or parallel relaxation. The greater the relaxation of the material, the better its behavior as a contrast agent should be, without taking into consideration biological factors that can alter its behavior, such as due to issues pertaining to their stability, aggregation, low cell internalization dose, or incomplete clearance. To improve the efficiency of T_2_ contrast agents, it is necessary to control the magnetic properties of the NPs through the design of the physical chemical properties, such as composition, crystalline structure, shape, hydrodynamic size, surface charge, and the nature of the coating. These characteristics also affect the biodistribution, stability, opsonization, and metabolism. Among all these physicochemical parameters, only three magnetic characteristics directly determine the T_2_ relaxivity: (1) saturation of magnetization value, (2) magnetic material volume fraction, (3) the intra-aggregate volume fraction occupied by the magnetic materials relative to the whole (hydrodynamic) sphere [5].

Previous reports highlight the relationship between the size of the NPs, the saturation magnetization (M_s_), and the r_2_ values. The larger the size and the M_s_ value, the higher the r_2_ relaxivity of the NPs [6]. Changes in the magnetic properties of the NPs are also observed depending on the synthesis method used [7,8]. These differences are mainly attributed to changes in the crystallographic order of the material or the presence of impurities. Another approach widely described in the literature is the modification of the contrast efficiency by tuning the composition and structure of the organic coating [9,10,11]. Although most commercial contrast agents are Gd-based, some other compounds based on iron oxides have come to be commercialized, such as Resovist, Lumirem etc., but their production was abandoned after a few years, due to toxicity and adverse effect problems [12]. Another strategy, based on the doping of iron oxide NPs with cations with atomic radii similar to Fe^2+^, such as Co, Ni, Mn, and Zn, has been carried out [13,14,15]. Taking into account an inversion degree of 100% in the spinel structure, AB_2_O_4_, this means that all the doped elements are placed in the B position, and the magnetic moment per formula unit corresponds to the μ_B_ of the doped cation. Considering the spin quantum numbers of Mn^2+^, Ni^2+^, and Co^2+^ cations, which correspond to S = 5/2, 3/2, and 1, respectively, the equivalent number of μ_B_ for paramagnetic transition metal cations can be estimated as follows: $\mu_{B}=2\cdot \sqrt{S\cdot \left(S+1\right)}$. Those magnetic moments correspond to the maximum theoretical values of 5.9 μ_B_, 3.9 μ_B_, and 2.8 μ_B_ for MnFe_2_O_4_, NiFe_2_O_4_, and CoFe_2_O_4_ ferrite structures. However, in the nanometric scale, the saturation magnetization is always lower than the bulk magnetization due to magnetic disorder of the surface. In any case, these ferrites show good performance as T_2_ contrast agents. Some examples from the literature show r_2_ values of approximately 358 mM^−1^s^−1^ for manganese ferrites with an M_s_ value of 110 emug^−1^ (Mn+Fe). An r_2_ value of approximately 394 mM^−1^s^−1^ has already been published by our group for electrochemically synthetized Mn_0.5_Fe_2.5_O_4_, with an M_s_ value of 108 emug^−1^ (Fe + Mn) [16]. Values of r_2_ = 152 mM^−1^s^−1^ with M_s_ = 85 emug^−1^ (Fe + Ni) were found for nickel ferrite [17,18]. Finally, in the case of cobalt ferrite NPs, several works have been published concerning several factors. For example, the relationship between sizes, M_s_, and transverse relaxivity (r_2_) values has been evaluated for cobalt ferrite nanocubes (NCs) in the range of 6–25 nm [15]. Albino et al. recently published the effect of the Zn inclusion in the cobalt ferrite structure, in the r_2_ relaxometric value. They established an optimal Zn substitution of 0.4 in the spinel unit formula to obtain an r_2_ value about 500 mM^−1^s^−1^ for 8 nm nanoparticles [19]. An improved modification of cobalt ferrite contrast agent has been carried out, doping with europium and encapsulated into mesoporous silica [20]. Novel T_2_ contrast agent materials recently published are focused on carbon-coated paramagnetic dysprosium oxide (DYO@C) nanoparticles [21].

Optimization of the magnetic properties is not the only point to be fulfilled. The stability and in vivo biocompatibility are also a forefront in the design of our nanoparticle MRI contrast agents. The long-term stability of these particles without agglomeration or precipitation is an important requirement for almost any application of magnetic nanoparticles. Moreover, certain types of coatings could favor targeting to specific organs and, therefore, an increase in the accumulation of the contrast agent in the desired area [22,23]. Among other small molecules, folic acid is well known as a target molecule that recognizes folate receptors overexpressed in certain tumor cell lines [24].

Under physiological conditions, most NPs penetrate the cells by endocytosis. Most endocytic pathways include lysosomes, where the degradation of nanoparticles takes place and reduces the diameter depending on the nature of the coating. This size reduction diminishes the in vitro signal and therefore reduces the final T_2_ contrast signal [25,26]. Finally, the cytotoxicity and cellular uptake of NPs also depend on morphological parameters of the particles, such as size, shape, coating, and surface charge, as well as biological parameters, such as cell line type, particle concentration, medium composition, and temperature.

Here, we propose the electrosynthesis of cobalt ferrite nanoparticles conjugated with folic acid for the specific recognition of the folate receptor expressed at the surface of cancer cells (HeLa). We characterized the nanocomposite (NP-FA) using Fourier transform infrared spectroscopy (FTIR), dynamic light scattering (DLS), and thermogravimetric analysis (TGA). The saturation magnetization values of cobalt ferrite nanostructures were measured by a superconducting quantum interference device (SQUID). Afterwards, the cytotoxicity and cytoskeleton morphology were evaluated after nanoparticle incubation. Finally, in vitro MRI relaxivity measurements of HeLa cells internalized with ferrite nanostructures were investigated for application in biomedicine.

## 2. Results and Discussion

### 2.1. Nanoparticle Physicochemical Properties

**Figure 1:**
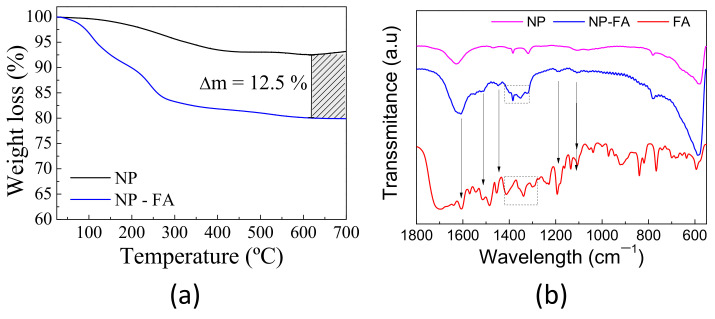
(**a**) Thermogravimetric analysis of bare nanoparticles (NP) (black line) and nanoparticle-folic acid (NP-FA) (blue line) under air conditions. The striped square is a guide for eyes to highlight the difference in weight loss between samples. (**b**) FTIR spectrum of bare NPs, NPs functionalized with folic acid, and the FA molecule.

Figure 1a shows the TGA data of bare NP and NP-FA. The non-functionalized sample exhibits a total weight loss of approximately 6.5% at 700 °C, and the weight decrease that appeared in the range of 100–400 °C was due to moisture removal and eventual tetrabutylammonium bromide evaporation. The NP-FA sample shows a total weight loss of 20% at 700 °C. Similar considerations as for bare NPs can be made; the steepest decrease between 200 and 500 °C could be due to the elimination of the covalently conjugated FA molecule. By considering the difference in moisture content between bare NP and NP-FA, we can estimate an experimental loss of folic acid of approximately 12%.

The surface groups of folic acid-modified CoFe_2_O_4_ nanoparticles were characterized by FTIR, as shown in Figure 1b. The absorption peak at 596 cm^−1^ is attributed to Fe-O bond absorption. Absorption at 1604 cm^−1^ corresponds to the carbonyl and aromatic ring stretching vibrations in the folic acid molecule, and absorption at 1396 cm^−1^ corresponds to the benzoic vibrations in the folic acid molecule. Therefore, infrared data proved that folic acid molecules successfully modified the surface of CoFe_2_O_4_ nanoparticles.

**Figure 2:**
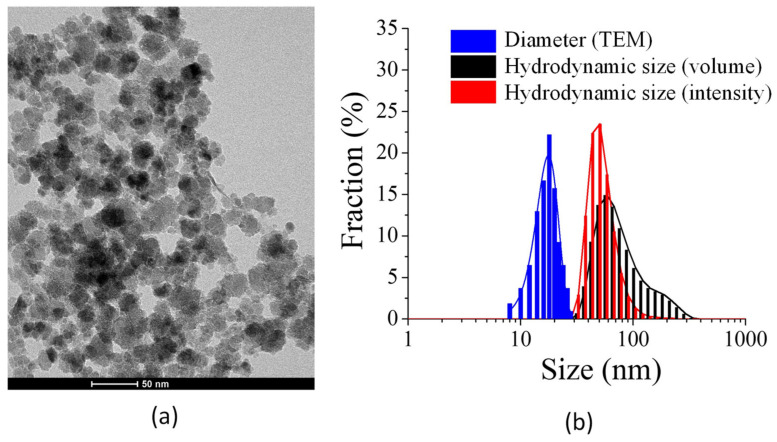
(**a**) Transmission electron microscopy (TEM) images of nanoparticles functionalized with folic acid. (**b**) Comparison of the nanoparticle size and hydrodynamic size distribution.

A selected TEM image for NP-FA is shown in Figure 2a. Processing of these TEM images by ImageJ software provided NP diameter values of 17(4) nm (N = 180). According to TEM, the functionalized nanoparticles present some aggregation.

The hydrodynamic diameter (HD) of dispersed NP-FA (the effective diameter of the NP-FA when diffusing in water, typically understood as the sum of the core diameter and twice the shell thickness) can be assessed with scattering techniques, e.g., dynamic light scattering (DLS). In Figure 2b, a comparison of the HD and the particle size is presented. The HD of the colloidal NP-FA sample was higher than the particle size, with a mean value of 78 nm (measured in intensity mode) and a polydispersity index (PDI) of 0.16. A PDI value < 0.2 reflects the size homogeneity of the sample [27].

One essential parameter concerning the behavior of magnetic nanoparticles to act as a T_2_ contrast agent is their saturation of magnetization (M_s_). A high M_s_ of superparamagnetic NPs locally induces higher magnetic field gradients if dispersed in solutions and subjected to an external homogeneous magnetic field. Thus, the higher the M_s_ of NPs with everything else being equal, the more effective they are as contrast agents.

**Figure 3:**
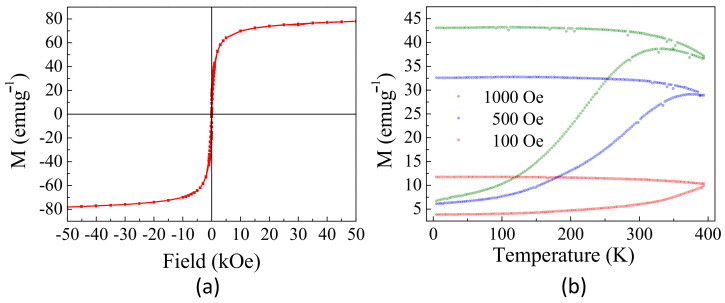
(**a**) Magnetic hysteresis loop at 300 K, (**b**) zero-field-cooling/field-cooling (ZFC/FC) curves, both of them for the sample NP-FA. Magnetization (M) was reflected as emu per gram of nanoparticle.

Figure 3a shows the hysteresis loops, M(H), measured up to 50 kOe at 300 K. At this temperature, an S-type curve with a hysteretic curve appears with H_c_ = 60 Oe, M_r_ = 11 emug^−1^, and an M_s_ value of 80.6(2) emug^−1^. This last value is close to the bulk value previously reported (80 emug^−1^) and higher than other values reported for cobalt ferrite nanoparticles with similar sizes and compositions [28,29].

The zero-field-cooling (ZFC) and field-cooling (FC) magnetization curves, from 5 to 395 K, at several external magnetic fields are depicted in Figure 3b. The FC curves show a nearly constant value of magnetization that decreases at high temperature and high magnetic fields, whereas the ZFC curves show a high irreversibility that decreases with the field. This behavior is related to ferro- or ferrimagnetic materials at the nanoscale with magnetic order temperatures above room temperature. All curves show similar behavior with a maximum in the ZFC curve at T_B_, the temperature related to the freezing or blocking process of the nanoparticle magnetic moment. As shown, the blocking temperature decreases with the external magnetic field, and a maximum is only present at approximately 335 K when working at 1000 Oe. The evolution of T_B_ with the magnetic field is related to the strong exchange interaction between the particles that allows a low-temperature order [30].

This finding indicates that below this temperature, NP-FA exhibits ferromagnetic behavior with remanence and coercivity and that above 300 K, the hysteresis feature vanishes and is expected to exhibit superparamagnetic behavior.

### 2.2. Relaxivity of NP-FA

**Figure 4:**
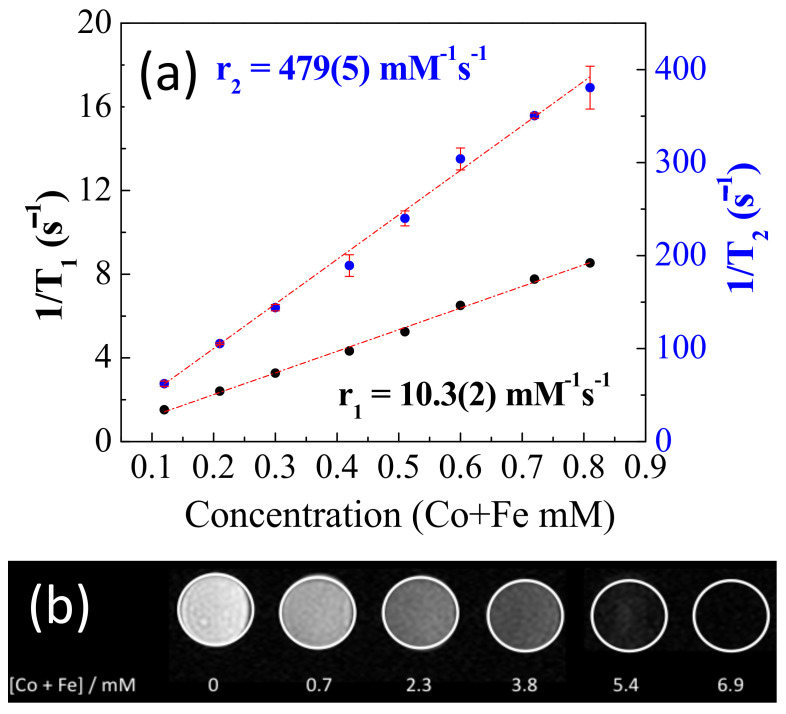
(**a**) T_1_ and T_2_ inverse measurements vs. cobalt and iron millimolar concentration. The slope of these curves corresponds to r_1_ and r_2_ relaxivities. (**b**) T_2_-weigthed magnetic resonance (MR) images of NP-FA in aqueous solution at various metal concentrations using a Varian 7T micro magnetic resonance imaging (MRI) scanner.

We then measured the MRI relaxation times (T_1_ and T_2_) from 0 to 0.8 mM iron and cobalt to obtain the transverse relaxivity (r_2_) and longitudinal relaxivity values (r_1_) of NP-FA, as shown in Figure 4a. The r_1_ value of NP-FA was approximately 10.3 Fe+Co mM^−1^s^−1^(2), whereas the r_2_ value was approximately 479(5) Fe+Co mM^−1^ s^−1^, providing an r_2_/r_1_ ratio of 48.3. The large r_2_ and r_2_/r_1_ ratios indicate that these nanoparticles are promising candidates for high-efficiency T_2_ contrast agents. The relaxation rate for zero metal concentration were extracted from the linear fitting of the experimental data, resulting in values of 0.2(1) s^−1^ and 4(2) s^−1^ for (1/T_1_)^0^ and (1/T_2_)^0^, respectively. Such a significant improvement in the T_2_ MR signal arises from the high value of saturation of magnetization of these NPs, which is able to distort the local magnetic field in an effective way. r_2_ is related to the M_s_, which, in turn, is affected by the size of the NP [11]. T_2_-weighted phantom images of a colloidal solution of NP-FA with different Fe and Co metal concentrations are shown in Figure 4b. With increasing concentration, the signal intensity of T_2_-weighted phantom images obviously decreases, as shown in Figure 4b, indicating that the T_2_ relaxation time decreases with increasing metal concentration. Therefore, the sample has the potential to generate MRI contrast enhancement on T_2_-weighted sequences.

Table 1 depicts the relaxivity values of most typical commercial agents based on iron oxide nanoparticles. For a simple comparison, several r_2_ values for cobalt ferrite nanoparticles found in the literature have also been summarized. The r_2_ value obtained in this work of cobalt ferrite nanoparticles functionalized with folic acid is the highest value found in the literature for nanoparticles with spherical shapes.

### 2.3. Cell Viability and Internalization

**Figure 5:**
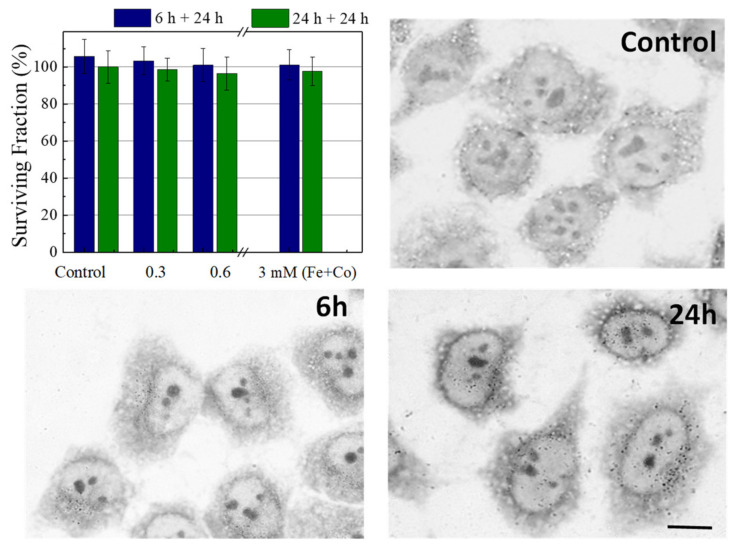
Bars diagram of biocompatibility of NPs functionalized with folic acid in HeLa cells measured by MTT assay. Blue columns, cells incubated 6 h with different concentrations of NPs and their viability measured 24 h later. Green columns, cells incubated for 24 h and their viability measured 24 h later. The results represent the average of 6 independent experiments. Optical micrographies of control cells, NP-FA 0.6 mM internalization in HeLa cells incubated for 6 and 24 h. Scale bar 10 µm.

The results obtained with the MTT assay do not exhibit any toxic effect for the concentrations used in this study. All viability percentages were similar to the controls, i.e., near 100% after 6 or 24 h of incubation, as shown in Figure 5.

After both incubation times (6 or 24 h), the cells were fixed and stained with toluidine blue (TB) and observed by optical microscopy, as shown in Figure 5. After 6 h of incubation at 0.6 mM, the NPs were clearly localized inside the cells and were detected as small aggregates, a control cell micrograph was shown for comparison. Moreover, the quantity of NPs inside the cells was higher if the incubation time was increased to 24 h. In addition, the NPs were kept inside the cells 24 h after the incubation time (data not shown). Reported toxicity investigations have revealed that the CoFe_2_O_4_ nanoparticles with appropriate surface modification show relatively low cytotoxicity [35,36].

### 2.4. Analysis of Cytoskeleton Components

Two cytoskeletal components, microtubules (MTs) and actin microfilaments, have also been analyzed to study the cytotoxic effect of NPs. Microtubules are highly dynamic fibers of the cytoskeleton organized as a radial array around the centrosome with critical functions in eukaryotic cells (e.g., motility, division, vesicle transport). Instead, actin microfilaments are involved in a wide variety of cell functions, such as the establishment of cell morphology, transport of vesicles and organelles, positioning of cellular components, cytokinesis, cell locomotion, cell–cell and cell–substrate interactions, and signal transduction.

**Figure 6:**
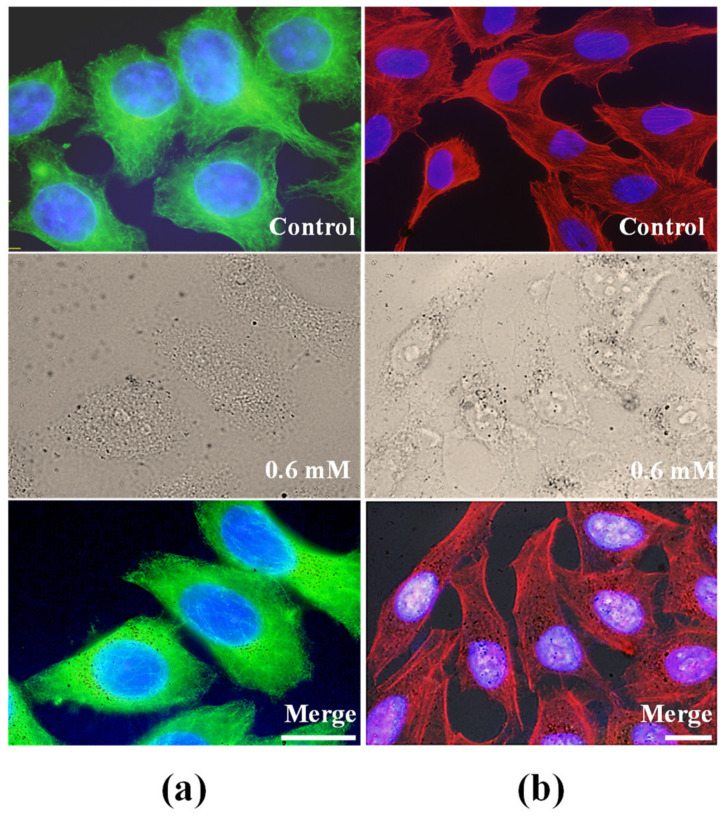
Analysis of cytoskeleton in HeLa cells incubated 24 h with 0.6 mM metal concentration and observed under fluorescence and bright-field microscopy immediately after incubations. (**a**) Immunofluorescence staining of α-tubulin observed under blue light excitation. (**b**) TRITC-phalloidin visualization of actin microfilaments observed under green light excitation.

We analyzed the effect of nanoparticle internalization on MTs and actin by indirect immunofluorescence analysis of α-tubulin and TRITC-rhodamine, respectively, and DNA counterstaining with Hoechst 33258. The interphase microtubule network appeared well organized in cells incubated for 24 h at a metal concentration of 0.6 mM compared with HeLa control cells, as shown in Figure 6a. Figure 6a also shows that the amount of NPs inside the cells (merged image) did not alter the microtubule morphology and distribution. To obtain more insight into possible indirect cell damage by these NPs, we also investigated their effects on actin microfilaments. As shown in Figure 6b, control cells showed stress fibers (red) crossing the cytoplasm, and under the cell cortex, they were anchored to the plasma membrane at focal contacts. In comparison, morphologic analysis of actin microfilaments after incubation with NPs did not show any alteration of this component under our experimental conditions, as shown in the merged image in Figure 6b.

In conclusion, the behavior of NPs in HeLa cells demonstrated easy NP penetration into cells under our experimental conditions, as well as the absence of effects on cell survival. As shown in several figures of this work, the morphology of cells incubated with NPs did not differ from that of control cells. The results obtained in the morphological analysis of the cytoskeletal components confirm the biocompatibility of the NPs.

### 2.5. In Vitro Phantom Image Contrast

**Figure 7:**
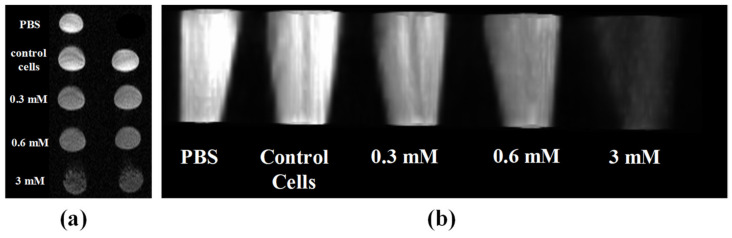
MRI of phantoms were performed to measure the T_2_ relaxivity of the SPION complex-labeled cells. (**a**,**b**) Transversal and longitudinal T_2_-weighted images of HeLa cells incubated with varied concentration of NP-FA.

The MR images from in vitro phantoms of NP-FA with different metal concentrations are shown in Figure 7. T_2_-weighted phantom MRI images were obtained from a series of colloidal suspensions of cells incubated with NP-FA in the transverse and longitudinal positions, as shown in Figure 7a,b, respectively. With increasing concentrations of NP-FA, the signal intensity dropped, resulting in a significant increase in T_2_ relaxation compared with phosphate-buffered saline (PBS) and control cells [11]. This suggests that a minimal concentration of NP-FA of 0.6 mM would be sufficient to detect an appreciably intense signal on MRI. In conclusion, the T_2_-weighted phantom images exhibited negative dose-dependent contrast improvement, suggesting that cobalt ferrite functionalized with FA as a target molecule is very promising for imaging purposes.

## 3. Materials and Methods

### 3.1. Synthesis and Functionalization of Cobalt Ferrite Nanoparticles

Cobalt ferrite nanoparticles were prepared in one step by an electrochemical method [37,38]. The grafting of the NPs with folic acid (FA) was performed by adding the as-synthetized NPs in an aqueous solution of 0.02 gmL^−1^ FA. The pH of this solution was increased by means of KOH until pH 8.0, and this solution was heated and stirred for 1 h at 80 °C. The NPs were separated by means of a magnet of 0.6 T and washed several times with distilled water to remove the excess FA, after which the pH of the aqueous solution was decreased to 7.2 with HNO_3_, and colloidal suspensions of particles were directly obtained by simple ultrasonic treatment. Finally, the suspension was dialyzed for 24 h to remove the FA not physi-absorbed to the nanoparticle surface.

### 3.2. Characterization Techniques

Electron transmission microscopy images were obtained after placing a single drop (10 μL) of the aqueous solution of NP-FA onto a copper grid coated with a carbon film. The grid was left to dry in air for several hours at room temperature. TEM analysis was carried out using a Tecnai T20 (FEI, Netherlands) electron microscope working at 200 kV. Magnetic characterization was carried out using a SQUID magnetometer (Quantum Design MPMS XL-5). The magnetization curves were measured at room temperature after applying a maximum magnetic field of 50 kOe. From the magnetization curves, values of parameters such as coercivity (H_c_) and saturation magnetization (M_s_) were obtained. The saturation magnetization was calculated by infinite field extrapolation of the experimental data obtained in the high field range where the magnetization linearly decreases with 1/*H*. The magnetization versus temperature curves were measured in zero-field-cooling (ZFC) and field-cooling (FC) procedures under different external applied magnetic fields, H = 100, 200, and 1000 Oe and between 5 and 395 K.

Thermogravimetric analysis (TGA) was conducted using a Mettler Toledo Instrument (TG/SDTA851e model) under air and with a DSC/821e model with a heating rate of 10 °C min^−1^ from room temperature to 800 °C. The presence of organic molecules attached to the nanoparticle surface was studied by infrared spectroscopy in a Nicolet 20 SXC FTIR. CoFe_2_O_4_–FA NPs were dispersed in KBr at 2 wt % and pressed in a pellet. The IR spectra were registered between 4000 and 300 cm^−^^1^.

Dynamic light scattering (DLS) measurements were carried out at 25 °C with a Nano ZS (Malvern Instruments) equipped with a solid-state He-Ne laser (λ = 633 nm) to determine the hydrodynamic diameter of the water dispersible NPs. The refraction index was 2.42 and the absorption 0.8. Finally, Fe and Co metal concentrations were measured with a Perkin-Elmer Optima 2100 DV inductively coupled plasma optical emission spectrometer (ICP-OES). For this purpose, samples were digested with nitric acid to oxidize the organic coating and then with hydrochloric acid to dissolve the particles.

### 3.3. Biological Characterization

#### 3.3.1. Cell Culture

Human carcinoma HeLa cells were grown in Dulbecco’s modified Eagle’s medium (DMEM) with 50 units mL^−1^ penicillin and 50 µg mL^−1^ streptomycin and supplemented with fetal bovine serum (FBS) at a concentration of 10% (whole medium). All media, serum, and antibiotics were provided by Invitrogen (Paisley, Scotland, UK). Cell cultures were performed in an incubator with a 5% CO_2_ atmosphere at 37 °C. The cells were seeded on 24-well multiwell dishes (Falcon, St. Louis, MO, USA) with or without coverslips. Experiments were performed with cells at 60–70% confluence.

#### 3.3.2. Nanoparticles

In vitro studies were performed using a 28 mM metal (Co + Fe) concentration stock solution of sonicated NP-FA, filtered under sterile conditions through a Millipore filter, aliquoted, and stored at 4 °C. Before use, the aliquots were sonicated again. Working solutions were adjusted to the desired concentrations in the whole medium.

#### 3.3.3. Biocompatibility Assay

A series of 24-well plates containing an identical number of cells were incubated for 6 or 24 h with solutions of 0.3, 0.6, and 3 mM metal, washed three times with sterile phosphate-buffered saline (PBS), and changed to whole medium. After 24 h, the medium was removed, and the cells were incubated with 3-(4,5-dimetil-tiazol-2-il)-2,5-difeniltetrazolio (MTT). Stock MTT solution (1 mgmL^−1^) was prepared in PBS and further diluted in complete medium to a final concentration of 0.05 mgmL^−1^. This solution was added to each well for 3 h at 37 °C. MTT (yellow and water soluble) is a compound transformed by cellular metabolism into formazan (blue and water insoluble). The amount of formazan produced is proportional to the number of living cells present in the culture. After 3 h of incubation, the medium was removed, and formazan was dissolved in dimethylsulfoxide (DMSO) and added to each well. The optical density was evaluated in a Tecan Spectra Fluor spectrophotometer (Männedorf, Switzerland) microplate reader at 540 nm. Considering that the NPs could affect the results, the DMSO solution was removed, and the absorbance of the plates was remeasured. The absorption value of formazan was obtained by subtracting the NP absorbance from the absorbance obtained in the first measurement (formazan + NPs). Cell survival was expressed as the percentage of absorption of treated cells relative to control cells. The mean value and standard deviation from at least six experiments were obtained.

#### 3.3.4. Internalization

To visualize the uptake of NPs, after the different experimental procedures, HeLa cells grown on coverslips were incubated in the presence of NP-FA (0.6 mM), washed with PBS (×5), fixed in cold methanol (5 min), stained with toluidine blue (TB Merck, Darmstadt, Germany) 0.05 mgmL^−1^ in distilled water for 30 s, washed with distilled water, and air dried. Preparations were mounted in DePeX (Serva, Heidelberg, Germany) and observed under brightfield microscopy.

#### 3.3.5. Cytoskeleton Analysis

Immunofluorescence was carried out in cells grown on coverslips to detect α-tubulin. HeLa cells were fixed in cold methanol for 5 min, washed 3 times for 5 min with PBS, permeabilized for 5 min with 0.5% Triton X-100 (*v*/*v*), and later incubated with the primary monoclonal mouse anti-α-tubulin antibody (1:100 in PBS/Bovine serum albumin(BSA), (Sigma, Darmstadt, Germany) for 1 h at 37 °C in a humidified chamber. After washing with PBS three times for 5 min, cells were incubated with the secondary goat anti-mouse antibody fluorescein isothiocyanate (FITC, from Sigma) at a dilution of 1:100 for 1 h at 37 °C. Finally, the cells were washed 3 times for 5 min with PBS, counterstained with the fluorochrome for DNA Hoechst-33258 (H-33258, Sigma) at a concentration of 0.6 mM in distilled water, washed with distilled water, and mounted in ProLong Gold antifade reagent (Molecular Probes). The fluorescence of FITC (green) and H-33258 (blue) was observed with a fluorescence microscope using the appropriate excitation filters.

For F-actin visualization, cells grown on coverslips were fixed in 3% paraformaldehyde for 10 min, washed in PBS for 15 min, and permeabilized with 0.5% (*v*/*v*) Triton X-100 in PBS for 20 min at room temperature. Cells were incubated with TRITC-phalloidin (Sigma) for 30 min at 37 °C, washed in PBS and counterstained with 5 µgmL^−1^ H-33258 in distilled water, washed with distilled water, and mounted in ProLong Gold. Samples were observed by fluorescence microscopy using the corresponding excitation filters.

#### 3.3.6. Confocal Microscopy

Microscopic observations were made, and photographs were taken with an Olympus BX61 fluorescence microscope equipped with ultraviolet (365 nm), blue (420 nm), and green (545 nm) filter sets for fluorescence microscopy and an Olympus DP50 Micromax digital camera from Princeton. Photographs were processed using Adobe Photoshop 8.0 (Adobe Systems, Adobe Systems, San Jose, CA, USA).

### 3.4. Relaxometric Characterization

#### 3.4.1. Magnetic Relaxivity Measurements

The ^1^H NMR relaxation times T_1_ and T_2_ were measured at 1.41 T in a Relaxometer Minispec mq-one analyzer from Bruker. The samples were prepared in distilled water and incubated at 37 °C for measurement. The concentrations used were less than 1 mM [Co + Fe]. T_1_ and T_2_ values were determined by the inversion−recovery method and by the Carr−Purcell−Meiboom−Gill sequence, respectively. Relaxivities (r_1_, r_2_) were obtained from the slopes of the curves 1/T_1_ (s^−1^) or 1/T_2_ (s^−1^) vs. the concentration of Co+Fe expressed in mM.

#### 3.4.2. Magnetic Resonance Imaging

MRI in phantoms and cells was performed with an Agilent/Varian scanner (Agilent, Santa Clara, CA, USA) equipped with a DD2 console and an active-shielded 205/120 gradient insert coil with 130 mTm^−1^ maximum gradient strength and a combination of volume coil/two-channel phased-array (Rapid Biomedical GmbH, Rimpar, Germany). Images were acquired using a gradient echo sequence with 4 ms/40 ms echo/repetition times, a Band Width (BW) of 100 kHz, and an Field of view (FOV) of 6 cm × 6 cm for a total acquisition time of approximately 80 s; the flip angle was fixed at 20 degrees.

## 4. Conclusions

We have demonstrated that the electrochemical method gives rise to stoichiometric cobalt ferrite nanoparticles with good magnetic properties and high relaxativity r_2_ values. The NPs were vectorized with folic acid to render a high uptake dose in the HeLa cancer cell line without a decrease in cell survival. The cytoskeleton retains its structure and morphology when nanoparticles are internalized at high doses. Finally, phantom T_2_-weighted images present a significant negative enhancement at low metal concentration doses.

We have demonstrated that the electrochemical method gives rise to stoichiometric cobalt ferrite nanoparticles in an easy and reproducible way, with good magnetic properties and some aggregation degree. The NPs were vectorized with folic acid to render a high uptake dose in the HeLa cancer cell line without a decrease in cell survival. MTT analysis reveals an almost null toxicity up to 3 mM of Co + Fe concentration. In addition, this low toxicity results in the cytoskeleton retaining its structure and morphology when nanoparticles are internalized at high doses. Relaxivity measurements of the NP-FA colloidal solution up to concentrations of 1 mM result in a high r_2_ value of 479 mM^−1^s^−1^. Finally, in vitro analysis with the HeLa cell line of phantom T2-weighted images present a progressive negative enhancement with the increase of concentration dose, in accordance with the high r_2_ value obtained.

## Figures and Tables

**Table 1:** Relaxivity collection values found in the literature as a function of nanoparticle diameter and coating.

Contrast Agents [31]	Material	Coating	D_(TEM)_ nm	r_1_	r_2_	r_2_/r_1_
This work	CoFe_2_O_4_	Folic acid	17(4)	10.3	479	46
Lee et al. [17]		2,3-Dimercaptosuccinic acid			172	
Schultz-Sikma et al. [32]		Silica	7		142	
Joshi et al. [15]		11-aminoundecanoic acid	15		301	
Kim et al. [18]		DMSA	8	6	392	62
Venkatesha et al. [33]		Chitosan	6		32	
Liu et al. [34]		Citrate	37.89	27.8	75.1	2.7
Sinerem^®^	γ-Fe_2_O_3_	Dextran	4–15	9.9	65	7
Resovist^®^		Carboxydextran	4–15	9.7	189	19
VSOP_C184^®^		Citric acid	5	14	33.4	2
Endorem^®^		Dextran	4–15	10.1	120	12

## Data Availability

The data presented in this study are available on request from the corresponding author.
